# Longitudinal Association of Coffee and Tea Consumption with Bone Mineral Density in Older Women: A 10-Year Repeated-Measures Analysis in the Study of Osteoporotic Fractures

**DOI:** 10.3390/nu17233660

**Published:** 2025-11-23

**Authors:** Ryan Yan Liu, Enwu Liu

**Affiliations:** College of Medicine and Public Health, Flinders University, Adelaide 5042, Australia; ryan.liu@flinders.edu.au

**Keywords:** bone mineral density (BMD), osteoporosis, coffee, tea, caffeine, postmenopausal women

## Abstract

**Background/Objectives**: Evidence regarding the associations between coffee and tea consumption and bone mineral density (BMD) in postmenopausal women remains inconclusive. Prior studies have not examined these relationships using repeated measures of both beverage intake and BMD over an extended follow-up. This study aimed to evaluate the longitudinal associations of coffee and tea consumption with BMD in older women. **Methods**: Data were drawn from the Study of Osteoporotic Fractures (SOF), a prospective cohort of 9704 women aged ≥65 years. Coffee and tea intake were repeatedly assessed via self-administered questionnaires at visits 2, 4, 5, and 6, spanning approximately 10 years. Femoral neck and total hip BMD were repeatedly measured by dual-energy X-ray absorptiometry. Linear mixed-effects models with random intercepts were used to estimate associations, adjusting for demographic, physical activity, comorbidities, and medication use. Nonlinear relationships were assessed using natural splines, and subgroup analyses were conducted using exposure-by-covariate interaction terms. **Results**: During the 10-year follow-up, tea consumption was positively associated with total hip BMD (least squares mean: 0.718 vs. 0.715 g/cm^2^; mean difference: 0.003; 95% CI: 0.000–0.005; *p* = 0.026). No significant overall association was observed on coffee consumption with femoral neck or total hip BMD. However, spline analyses suggested that consuming more than five cups of coffee per day may be associated with lower BMD. Interaction analyses indicated significant interactions between coffee and alcohol intake (*p* = 0.0147) and between tea consumption and BMI (*p* = 0.0175). **Conclusions**: Tea consumption was associated with higher total hip BMD in postmenopausal women, whereas excessive coffee intake (>5 cups/day) may adversely affect BMD. Coffee consumption was negatively associated with femoral neck BMD in women with higher alcohol intake, while tea consumption appeared particularly beneficial for those with obesity.

## 1. Introduction

Osteoporosis, characterized by low bone mineral density (BMD), is a major global public health concern affecting individuals across all ages, genders, and ethnicities [1,2]. Worldwide, approximately one in three women and one in five men aged 50 years or older experience osteoporotic fractures [3]. According to the Global Burden of Disease Study 2021, low BMD accounted for 8.32 million years lived with disability (YLDs), 17.2 million disability-adjusted life years (DALYs), and 477,000 deaths, with 71% of these deaths directly attributable to low BMD [4]. In the United States, the number of adults aged 50 years and older with low BMD at the femoral neck or lumbar spine is projected to rise by 17.2 million by 2030 [5]. Fractures related to osteoporosis impose substantial morbidity, health care costs, and long-term disability burdens [6]. Women are particularly vulnerable due to smaller bone mass and the accelerated bone loss that occurs after menopause [7].

Dietary factors play a crucial role in the prevention of noncommunicable diseases and may influence bone health [8]. Coffee and tea are among the most widely consumed beverages globally. The International Coffee Organization reported the consumption of 177 million bags of coffee (1 bag = 60 kg) in 2023–2024 [9], while global tea consumption has reached approximately 6.5 million metric tons annually [10]. Caffeine, a major bioactive compound in both beverages, has been the focus of numerous studies investigating its potential effects on health outcomes, including bone metabolism [11,12]. However, evidence regarding the relationship between coffee and tea consumption and BMD remains inconsistent and none has employed repeated measurements of both exposures and outcomes over an extended follow-up period.

This study aimed to examine the longitudinal associations between coffee and tea consumption and BMD among postmenopausal women, using repeated-measures data from the Study of Osteoporotic Fractures (SOF).

## 2. Materials and Methods

### 2.1. Data Source

The study data were drawn from the SOF, a multicenter prospective cohort study that followed 9704 primarily White women aged ≥65 years for approximately 20 years, with clinical assessments conducted every two years (nine visits in total). The detailed study design has been described elsewhere [13]. All participants provided written informed consent, and institutional review board approval was obtained. This specific analysis was exempted from ethical review by the Flinders University Human Research Ethics Committee (project number: 9307).

### 2.2. Study Population

The study population included SOF participants who attended visits 2, 4, 5, and 6, spanning approximately 10 years, during which BMD and coffee and tea consumption were both assessed. Data from visits 1, 3, and 7 were excluded due to the unavailability of either BMD or beverage intake data. Visits 8 and 9 were excluded from the primary analysis because of extensive missing data but were included in the sensitivity analyses.

### 2.3. Exposure

Exposures were assessed using self-administered questionnaires. The exposure-related questions were as follows: for coffee consumption, “Do you currently drink regular coffee? (yes/no); if yes, on average, how many cups of regular coffee do you usually consume per day?”; for tea consumption, “Do you currently drink tea or iced tea (excluding herbal or decaffeinated varieties)? (yes/no); if yes, on average, how many cups of tea do you usually consume per day?”.

### 2.4. Outcome

The outcome variables were femoral neck BMD (g/cm^2^) and total hip BMD (g/cm^2^), measured at visits 2, 4, 5, and 6 using a Hologic QDR 1000 densitometer, where visit 6 was treated as the primary endpoint. These anatomical sites were selected because of their strong correlation with fracture risk [14,15].

### 2.5. Covariates

Time-dependent covariates included: visit, age, body mass index (BMI, kg/m^2^), current cigarette smoking status (yes/no), and the Charlson comorbidity index (CCI), derived from participants’ medical histories. The detailed algorithm for CCI calculation has been described previously [16].

Time-independent covariates were primarily collected at visit 1 and included age at menopause, lifetime alcohol intake (calculated as drinks per week multiplied by years of drinking), lifetime physical activity (a weighted measure of frequency across four life stages: teen, 30s, 50s, and current), oral estrogen use, and oral steroid pill use (both categorized as current, past, or never). Average weekly calcium and protein intake were both assessed at visit 1 using a 20-item dietary scale. Finally, baseline BMD measured at visit 2 was included as a time-independent covariate specific to each corresponding outcome site.

### 2.6. Statistical Analysis

Coffee and tea consumption, along with covariates measured at each visit, were treated as categorical or continuous variables and are presented as frequencies (percentages) or means (standard deviations), respectively.

The associations between coffee or tea consumption and BMD were assessed using linear mixed-effects models with random intercepts [17]. Two models were specified. Model 1 (minimally adjusted): included the outcome variable (femoral neck or total hip BMD), the primary exposure (coffee or tea consumption), the study visit, the visit × exposure interaction, and baseline BMD corresponding to the outcome site. Model 2 (extensively adjusted): additionally adjusted for time-dependent covariates (age, BMI, current smoking status, Charlson comorbidity index), the other beverage exposure (e.g., tea intake adjusted for coffee intake) and time-independent covariates (age at menopause, lifetime alcohol use, average weekly calcium and protein intake, weighted lifetime physical activity, and oral estrogen and steroid pill use).

Subgroup analyses were performed to evaluate if the associations between coffee or tea intake and BMD varied across participant characteristics. This was achieved by including two-way interaction terms between each exposure and the covariates in the mixed-effects models [18,19,20]. For continuous covariates, the variables were dichotomized at their respective medians for the interaction assessment. Least squares [LS] means and pairwise comparisons were estimated using the LSMEANS and SLICE statements in SAS.

To assess potential non-linear relationships, natural spline regression models were applied for continuous exposures (cups of coffee or tea per day) [21]. Based on previous research [22,23], two knots were placed at 2 and 4 cups per day to define points of interest. Non-linearity was assessed by a likelihood ratio test comparing the non-linear and linear models. Additionally, to ensure stable predictions, the following variables were square root transformed: lifetime alcohol use, average weekly calcium intake, average weekly protein intake, and weighted lifetime physical activity.

For the main analyses, missing data were assumed to be Missing at Random (MAR). No specific imputation procedures were applied, as linear mixed models can appropriately handle missing data under the MAR assumption [24,25].

Sensitivity analyses were conducted by extending the follow-up period to include visits 8 and 9. Natural spline regression models were also re-run using this extended dataset.

All statistical analyses were conducted using SAS software, version 9.4 (SAS Institute Inc., Cary, NC) and R software, version 4.4.0 (R Core Team, Vienna, Austria)

## 3. Results

**Figure 1:**
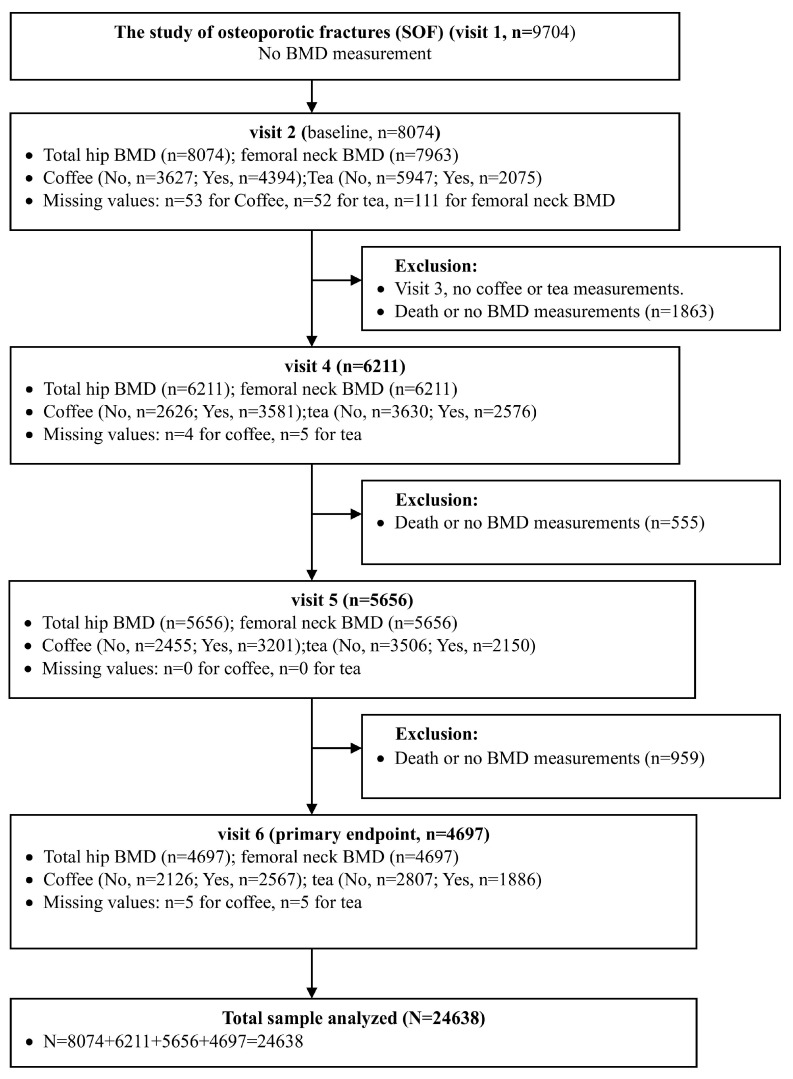
Study flow diagram.

The study flow diagram detailing participant inclusion is presented in Figure 1. A total of 9704 women were initially enrolled in the SOF at Visit 1; however, BMD was not measured at this time point. At visit 2 (baseline), 8074 participants had total hip BMD data available, and 7963 had femoral neck BMD data. Subsequent follow-up visits (visits 4, 5, and 6) included 6211, 5656 and 4697 participants, respectively, with both BMD and beverage consumption data collected at each visit. Participants who died or had missing BMD data were excluded from each subsequent wave. After all exclusions across the four included visits (visits 2, 4, 5, and 6), the final total analytic sample comprised 24,638 observations.

The mean follow-up period for the analytic sample was 8.1 ± 0.6 years, with a median of 8.0 years (interquartile range [IQR], 0.7 years). Table 1 summarizes the outcome variables and covariates by coffee and tea consumption across the four study visits (2, 4, 5, and 6).

Over the approximately 10-year follow-up period, femoral neck BMD declined from a mean of approximately 0.65 g/cm^2^ at visit 2 to 0.62 g/cm^2^ at visit 6. For total hip BMD, the mean value decreased from approximately 0.76 g/cm^2^ to about 0.73 g/cm^2^.

The mean age of participants increased from approximately 73 years at visit 2 to 80 years by visit 6. Mean BMI remained relatively stable at around 26 kg/m^2^ throughout follow-up. The CCI increased, rising from approximately 1.3 at visit 2 to 2.9 at visit 6.

Smoking prevalence decreased across all groups. Among non-coffee drinkers, it decreased from 5.4% to 2.4%, and among coffee drinkers, it decreased from 10.0% to 5.4%. Similarly, smoking prevalence declined from 8.3% to 4.5% among non-tea drinkers and from 6.7% to 3.4% among tea drinkers.

The mean age at menopause across all groups was approximately 48 years. Non-coffee drinkers and non-tea drinkers generally reported higher average weekly calcium consumption than their respective beverage-drinking counterparts, with the exception of the non-tea drinker group at visit 6. Coffee drinkers consumed slightly less protein than non-coffee drinkers, while tea drinkers consumed slightly more protein than non-tea drinkers. Coffee drinkers reported higher alcohol consumption than non-drinkers, whereas tea drinkers reported lower alcohol consumption than non-drinkers, except at visit 2. No clear pattern was observed in lifetime physical activity levels between coffee/tea drinkers and non-drinkers. At baseline, about 15% of women were current oral estrogen users, and less than 3% were current oral steroid pill users.

Table 2 presents the associations between coffee consumption and femoral neck and total hip BMD as estimated by the linear mixed-effects models. Across all four study visits, coffee consumption was not significantly associated with either femoral neck or total hip BMD in the minimally adjusted Model 1 or the extensively adjusted Model 2.

Table 3 details the associations between tea consumption and femoral neck and total hip BMD. Tea consumption showed no significant association with femoral neck BMD in either the minimally adjusted Model 1 or the extensively adjusted Model 2.

However, tea consumption was significantly associated with higher total hip BMD at Visit 6 in both models. In model 1, the association was observed with a LS mean difference of 0.002 (LS mean: 0.717 vs. 0.715; 95% CI: 0.000–0.004; *p* = 0.0354). This finding persisted in the extensively adjusted Model 2, which showed a slightly stronger association with an LS mean difference of 0.003 (LS mean: 0.718 vs. 0.715; 95% CI: 0.000–0.005; *p* = 0.0260).

**Figure 2:**
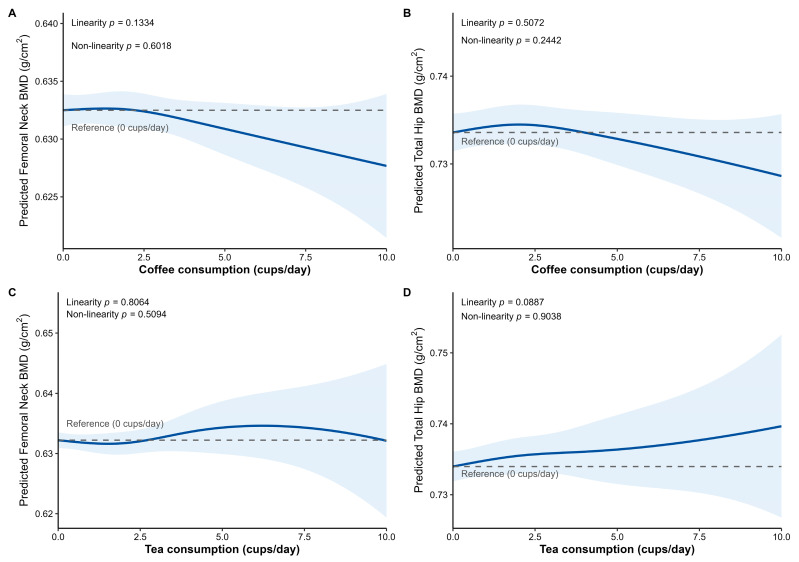
Association between coffee and tea consumption and femoral neck BMD and total hip BMD. (**A**) Coffee consumption and femoral neck BMD. (**B**) Coffee consumption and total hip BMD. (**C**) Tea consumption and femoral neck BMD. (**D**) Tea consumption and total hip BMD. Predicted values and their 95% confidence intervals for BMD were obtained from the extensively adjusted linear mixed-effects model utilizing natural splines for coffee or tea consumption (cups/day). The model included adjustment for time-dependent covariates (visit, age, BMI, smoking status, and CCI) and time-independent covariates (age at menopause, lifetime alcohol use, average weekly calcium and protein intake, weighted lifetime physical activity, oral estrogen use, oral steroid pill use, and baseline BMD corresponding to the outcome site). Two knots were placed at 2 and 4 cups per day for both coffee and tea consumption. The dashed line indicates the reference value for the predicted BMD at 0 cups/day, and the shaded blue area denotes the 95% confidence interval. The *p*-value for linearity was derived from the model treating coffee or tea consumption as a linear term, and the *p*-value for non-linearity was obtained by comparing the spline and linear models using a likelihood ratio test.

Figure 2 presents the results from natural spline regression analyses. The likelihood ratio tests indicated no significant non-linear associations between the continuous daily consumption of coffee or tea and either femoral neck or total hip BMD (*p* for nonlinearity all >0.05).

However, the trends suggested by Figure 2A,B indicate that consuming approximately 2 to 3 cups of coffee per day may not affect BMD. Conversely, consumption exceeding 5 cups per day was visually suggested to be potentially associated with lower BMD.

For tea consumption, a weak linear relationship may exist with total hip BMD (β = 0.0006, *p* for linearity = 0.0887; Figure 2D).

**Figure 3:**
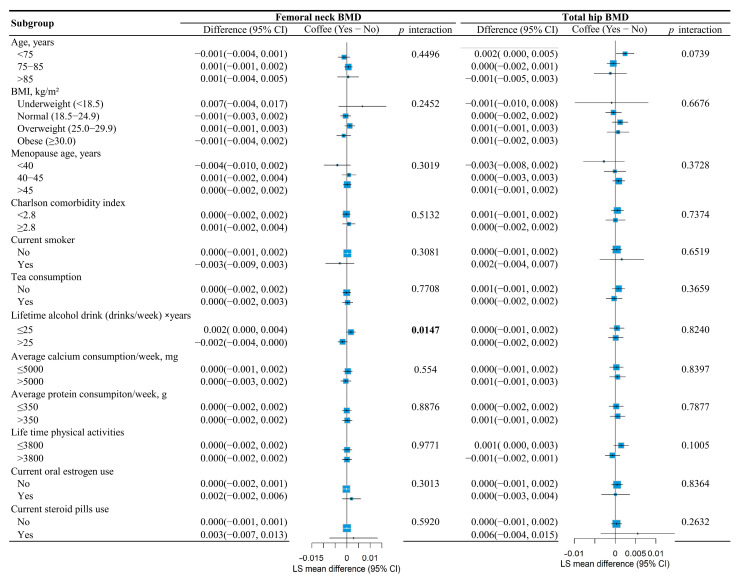
Subgroup analysis evaluating the association between coffee consumption and femoral neck BMD and total hip BMD among different subgroups through coffee × covariate interactions in the mixed models.

Subgroup analyses revealed significant interaction effects. The association between coffee consumption and femoral neck BMD was significantly modified by lifetime alcohol intake (*p* for interaction = 0.0147). Specifically, coffee consumption showed a more beneficial effect on femoral neck BMD among participants categorized as consuming less alcohol (LS mean difference = 0.002; 95% CI, 0.000 to 0.004), as illustrated in Figure 3.

**Figure 4:**
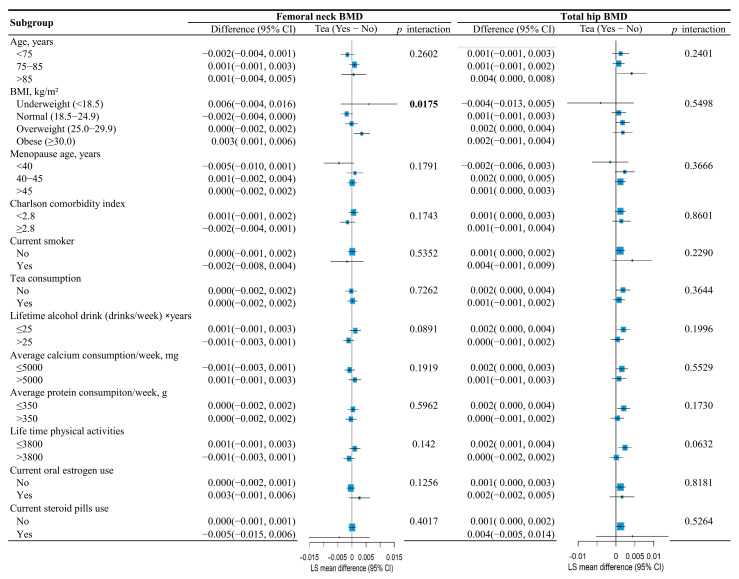
Subgroup analysis evaluating the association between tea consumption and femoral neck BMD and total hip BMD among different subgroups through tea × covariate interactions in the mixed models.

Furthermore, the association between tea consumption and femoral neck BMD was significantly modified by BMI (*p* for interaction = 0.0175). Tea consumption was associated with higher femoral neck BMD among women classified as having obesity (LS mean difference = 0.001; 95% CI, 0.001 to 0.006), as shown in Figure 4.

Sensitivity analyses, extending the follow-up period to include visits 8 and 9, provided additional insights. At visit 9, coffee consumption was associated with lower femoral neck BMD but showed no significant association with total hip BMD. Conversely, tea consumption was associated with higher total hip BMD at visits 6 and 8, but this association was not observed at visit 9 (Appendix A Table A1 and Table A2).

**Figure A1:**
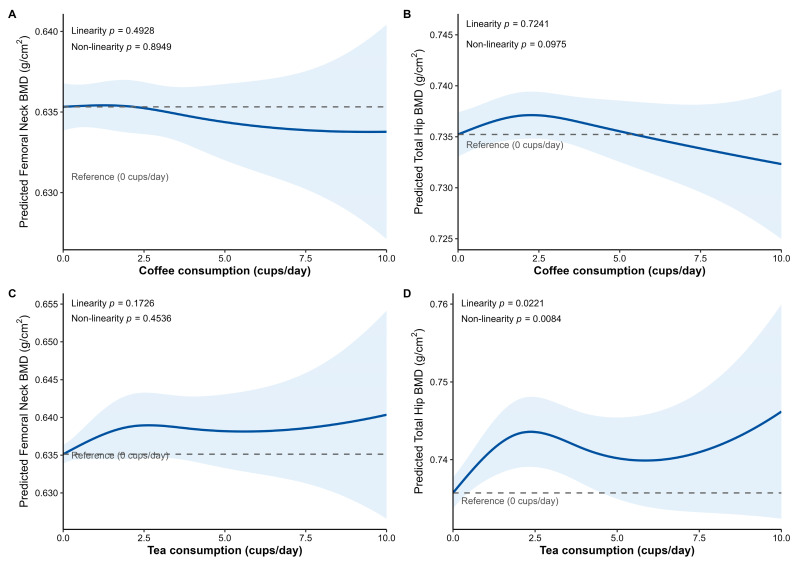
Association between coffee, tea consumption and femoral neck BMD and total hip BMD. (**A**) Coffee consumption and femoral neck BMD. (**B**) Coffee consumption and total hip BMD. (**C**) Tea consumption and femoral neck BMD. (**D**) Tea consumption and total hip BMD. Predicted values and 95% confidence intervals for BMD were obtained from a linear mixed-effects model with natural splines for coffee or tea consumption (cups/day), adjusted visit, age, BMI, menopausal age, smoking status, Charlson comorbidity index, and the other beverage, and time independent covariates age at menopause, lifetime alcohol use, average calcium consumed per week, average protein consumed per week, weighted lifetime physical activity, oral estrogen used, steroid pills use and baseline BMD. Two knots were placed at 2 and 4 cups per day for both coffee and tea consumption. The dashed line indicates the reference value at 0 cups/day, and the shaded blue area denotes the 95% confidence interval. The *p* value for linearity was obtained from the model including coffee or tea consumption as a linear term, and the *p* value for nonlinearity was obtained by comparing the spline and linear models using a likelihood ratio test.

For non-linear relationships, the natural spline regression conducted on the extended dataset visually confirmed the suggestion that consuming more than 5 cups of coffee per day might be associated with lower BMD. For tea consumption and total hip BMD, we observed a significant overall linear association (β = 0.001, *p* = 0.0221), alongside evidence that the association departs from linearity (likelihood ratio test *p* = 0.0084). This departure indicates additional curvature beyond the simple linear trend (Appendix A Figure A1).

## 4. Discussion

In this 10-year repeated-measures study of women aged ≥65 years, we found that tea consumption was positively associated with total hip BMD. While the statistical test for non-linearity was non-significant, our natural spline regression analyses visually suggested that consuming approximately two to three cups of coffee per day might not affect BMD, whereas intake exceeding five cups per day could be linked to lower BMD. Tea consumption showed a linear association with total hip BMD; however, the dose-response relationship was modest (β = 0.0006, *p* for linearity = 0.0887). Subgroup analyses suggested that women with higher lifetime alcohol intake may benefit from limiting coffee consumption, while women with obesity might derive greater benefits from tea consumption. However, these subgroup findings should be interpreted with caution as they are likely underpowered and may be susceptible to type I error; they are primarily hypothesis-generating and warrant validation in future, larger studies. The sensitivity analyses were largely consistent with our main results, although the finding that coffee consumption was associated with lower femoral neck BMD at visit 9 should be interpreted cautiously due to the substantial missing data at that late time point.

The relationship between coffee consumption and BMD remains inconsistent across the literature. Several studies have reported that higher coffee intake is associated with lower BMD [26,27,28,29], whereas others have found no association [30,31,32], and some have suggested a protective effect [33,34,35,36]. Caffeine, a key component of coffee, is believed to play a significant role in these mixed findings [37]. Mechanistically, caffeine acts as a non-specific antagonist of adenosine receptors, which may inhibit bone formation and enhance bone resorption [28]. In vitro studies further suggest that caffeine may downregulate vitamin D receptor expression and impair the osteogenic actions of 1,25(OH)_2_D_3_ in osteoblasts [38]. However human experimental studies indicate that in individuals with inadequate calcium intake, caffeine’s impact is minimal, causing only a slight negative calcium balance due to reduced absorption efficiency. This effect is often negligible that it could be offset by adding one to two tablespoons of milk to a cup of coffee [39]. Furthermore, genetic factors may modify the association between caffeine and osteoporosis risk. For example, a study using Taiwan Biobank data found that individuals carrying the ESR1 rs2982573 genotype who consumed more than three cups of coffee per week had a lower risk of osteoporosis [40].

Findings regarding tea consumption and BMD have also been inconsistent. Some studies, such as those conducted among Korean postmenopausal women and in China [41,42], found that habitual tea drinking was associated with higher BMD at various skeletal sites. Research in the UK among women aged 65 to 76 years [43], as well as studies in Australian women aged 70 to 85 years [44], reported similar positive associations. Two meta-analyses have also supported the potential benefits of tea consumption on BMD [45,46]. However, other studies, including a clinical trial of green tea extract, found no significant associations [47], and another meta-analysis concluded that tea consumption was not associated with higher BMD [31]. The potential mechanisms underlying tea’s effects may involve catechins, particularly epigallocatechin (EGC), which can promote osteoblast activity and inhibit osteoclast differentiation [48]. Animal studies also suggest that EGC may prevent both bone and muscle loss [49].

Our study offers several strengths, notably its basis in a large, well-characterized cohort with repeated measures of both beverage intake and BMD, which helps minimize measurement error. Furthermore, the use of linear mixed-effects models provides a robust analytic framework that effectively accounts for time-varying exposures and covariates, better reflecting real-world changes over time.

However, limitations must be considered. First, the self-reported nature of coffee and tea consumption may introduce measurement bias. In addition, treating intrinsically time-varying variables, such as physical activity and alcohol consumption, as time-independent, by only assessing lifetime consumption at baseline, may also introduce recall bias. Furthermore, more detailed information, such as cup size, beverage strength, and beverage types, was not collected, which may further contribute to measurement error. Second, the long follow-up period resulted in high rates of mortality and missing data. While mixed models can handle missingness under the assumption of MAR, this assumption may not fully hold, potentially introducing bias. Third, despite extensive covariate adjustment, residual confounding cannot be entirely ruled out. Fourth, although the difference in BMD between tea drinkers and non-drinkers (≈0.003 g/cm^2^) is statistically significant, it is below the threshold typically considered clinically meaningful for individual patient management [50], However, such a small difference may still be relevant from an epidemiological and public health perspective, as even modest shifts in the population distribution of BMD could translate into reductions in fracture risk at the group level. Future longitudinal studies with fracture as a primary endpoint are necessary to confirm whether the small BMD differences associated with tea consumption have a meaningful impact on reducing osteoporotic fractures. Finally, the study population consisted almost entirely of White older women in the United States. This lack of racial and ethnic diversity severely restricts the applicability of our findings to broader populations. Because BMD, fracture risk, and dietary patterns differ meaningfully across racial/ethnic groups, our results should be interpreted with considerable caution when applied outside this demographic.

## 5. Conclusions

Our findings suggest tea consumption is associated with higher total hip BMD in older postmenopausal women. While no overall association between coffee consumption and BMD was identified during the 10-year follow-up, natural spline analyses suggest that consuming more than five cups of coffee per day might be detrimental to BMD. The observed interaction effects highlight the importance of individual factors: coffee consumption was negatively associated with femoral neck BMD in women with higher alcohol intake, while tea consumption appeared particularly beneficial for those with obesity. These findings underscore the importance of personalized dietary recommendations in promoting optimal bone health among aging women.

## Figures and Tables

**Table 1:** Distribution of outcome variables and covariates by coffee and tea consumption across study visits.

Variable	Visit	Coffee		Tea	
		No	Yes	No	Yes
Femoral neck BMD, N, mean (SD), g/cm^2^	2	3577, 0.651 (0.109)	4334, 0.647 (0.112)	5865, 0.648 (0.111)	2047, 0.649 (0.109)
	4	2626, 0.634 (0.115)	3581, 0.630 (0.117)	3630, 0.631 (0.115)	2576, 0.634 (0.118)
	5	2455, 0.629 (0.116)	3201, 0.626 (0.119)	3506, 0.627 (0.119)	2150, 0.627 (0.117)
	6	2126, 0.625 (0.116)	2566, 0.623 (0.125)	2806, 0.626 (0.121)	1886, 0.621 (0.120)
Total hip BMD, N, mean (sd), g/cm^2^	2	3627, 0.760 (0.129)	4394, 0.754 (0.133)	5947, 0.757 (0.132)	2075, 0.757 (0.129)
	4	2626, 0.739 (0.133)	3581, 0.734 (0.134)	3630, 0.735 (0.134)	2576, 0.738 (0.133)
	5	2455, 0.733 (0.133)	3201, 0.728 (0.137)	3506, 0.729 (0.136)	2150, 0.732 (0.134)
	6	2126, 0.727 (0.133)	2566, 0.723 (0.141)	2806, 0.725 (0.139)	1886, 0.725 (0.136)
Age, N, mean (sd), y	2	3610, 73.4 (5.1)	4380, 73.2 (4.9)	5926, 73.3 (5.0)	2065, 73.3 (5.0)
	4	2622, 76.6 (4.7)	3576, 76.5 (4.6)	3624, 76.4 (4.6)	2573, 76.7 (4.7)
	5	2449, 78.3 (4.4)	3191, 78.3 (4.4)	3496, 78.2 (4.4)	2144, 78.4 (4.4)
	6	2121, 80.0 (4.1)	2564, 80.0 (4.1)	2802, 80.0 (4.1)	1883, 80.0 (4.1)
BMI, N, mean (sd), kg/m^2^	2	3516, 26.2 (4.4)	4278, 26.2 (4.4)	5786, 26.2 (4.4)	2009, 26.2 (4.4)
	4	2572, 26.5 (4.6)	3511, 26.4 (4.5)	3547, 26.3 (4.5)	2535, 26.6 (4.5)
	5	2419, 26.5 (4.6)	3154, 26.5 (4.6)	3453, 26.5 (4.6)	2120, 26.5 (4.6)
	6	2081, 26.5 (4.6)	2519, 26.3 (4.5)	2748, 26.4 (4.6)	1852, 26.4 (4.5)
Charlson comorbidity index, N, mean (sd)	2	3627, 1.4 (1.7)	4394, 1.3 (1.7)	5947, 1.3 (1.7)	2075, 1.3 (1.7)
	4	2626, 2.9 (2.8)	3581, 2.6 (2.8)	3630, 2.7 (2.8)	2576, 2.8 (2.8)
	5	2455, 3 (2.9)	3201, 2.8 (2.8)	3506, 2.9 (2.9)	2150, 2.9 (2.8)
	6	2126, 3.1 (2.9)	2567, 3 (2.9)	2807, 3 (2.9)	1886, 3.1 (2.9)
Current smoker, yes/total (%)	2	194/3627 (5.4)	439/4393 (10.0)	494/5946 (8.3)	139/2075 (6.7)
	4	95/2626 (3.6)	255/3581 (7.1)	238/3630 (6.6)	112/2576 (4.4)
	5	66/2455 (2.7)	202/3201 (6.3)	203/3506 (5.8)	65/2150 (3.0)
	6	51/2126 (2.4)	128/2567 (5.4)	125/2807 (4.5)	64/1886 (3.4)
Menopause age, mean (sd)	2	2930, 48.2 (5.6)	3630, 47.9 (5.8)	4871, 48.1 (5.7)	1690, 47.8 (5.8)
	4	2116, 48.2 (5.6)	2963, 48.1 (5.7)	2972, 48.1 (5.7)	2107, 48.2 (5.7)
	5	1984, 48.3 (5.5)	2633, 48.1 (5.7)	2847, 48.1 (5.6)	1770, 48.3 (5.5)
	6	1702, 48.5 (5.4)	2117, 48.2 (5.7)	2265, 48.3 (5.6)	1554, 48.3 (5.5)
Average calcium consumed per week (mg), N, median (IQR)	2	3627, 4422.7 (4009.6)	4394, 4320.8 (3624.4)	5947, 4394.1 (3947.3)	2075, 4325.4 (3480.5)
	4	2626, 4411.5 (4015.1)	3581, 4336.7 (3592.5)	3630, 4351.6 (3942.8)	2576, 4382.1 (3480.7)
	5	2455, 4360.3 (3909.3)	3201, 4334.4 (3659.7)	3506, 4334.4 (3887.5)	2150, 4370.5 (3594.6)
	6	2126, 4333 (3949.3)	2566, 4377.8 (3574.6)	2806, 4319.6 (3865.3)	1886, 4416.7 (3546.9)
Average protein consumed per week (g), N, median (IQR)	2	3627, 342.9 (171.9)	4394, 342.1 (172.6)	5947, 340.7 (171)	2075, 348 (173.6)
	4	2626, 344.2 (172.1)	3581, 340.9 (168.1)	3630, 339.1 (171.6)	2576, 345.5 (167.8)
	5	2455, 341.7 (174.4)	3201, 343.1 (167.4)	3506, 340.5 (174.7)	2150, 345.1 (166.3)
	6	2126, 341.2 (173.7)	2566, 343.4 (165.4)	2806, 338.7 (170.3)	1886, 347.7 (167.4)
Lifetime alcohol use (drinks/week) ×years, N, median (IQR)	2	3627, 13.8 (76.5)	4394, 28.1 (115.3)	5947, 21.0 (93.1)	2075, 26.8 (99.0)
	4	2626, 15.0 (80.0)	3581, 28.8 (112.3)	3630, 23.1 (95.5)	2576, 26.3 (100.0)
	5	2455, 15.4 (79.5)	3201, 29.4 (110.8)	3506, 23.1 (97.4)	2150, 26.9 (94.8)
	6	2126, 21.5 (85.4)	2566, 30.0 (108.3)	2806, 24.2 (96.3)	1886, 29.4 (97.8)
Number of physical activities per year over lifetime, N, median (IQR)	2	3520, 3830 (4400)	4268, 3670 (4227.5)	5769, 3750 (4350)	2020, 3690 (4165)
	4	2552, 3950 (4325)	3479, 3750 (4255)	3516, 3877.5 (4245)	2514, 3765 (4340)
	5	2389, 3810 (4255)	3106, 3852.5 (4185)	3398, 3910 (4190)	2097, 3745 (4255)
	6	2067, 3870 (4290)	2494, 3812.5 (4200)	2709, 3800 (4185)	1852, 3862.5 (4342.5)
Current oral estrogen use, yes/total (%)	2	529/3569 (14.8)	608/4346 (14)	856/5874 (14.6)	281/2042 (13.8)
	4	377/2579 (14.6)	535/3544 (15.1)	526/3574 (14.7)	386/2548 (15.1)
	5	357/2416 (14.8)	483/3167 (15.3)	520/3454 (15.1)	320/2129 (15.0)
	6	310/2094 (14.8)	406/2536 (16.0)	429/2773 (15.5)	287/1857 (15.5)
Current use steroid pills, yes/total (%)	2	74/3556 (2.1)	77/4320 (1.8)	101/5839 (1.7)	50/2038 (2.5)
	4	48/2572 (1.9)	52/3527 (1.5)	57/3566 (1.6)	43/2532 (1.7)
	5	39/2412 (1.6)	41/3147 (1.3)	54/3447 (1.6)	26/2112 (1.2)
	6	37/2087 (1.8)	29/2527 (1.1)	38/2767 (1.4)	28/1847 (1.5)

Abbreviations: N: sample size without missing values, BMI, body mass index.

**Table 2:** Associations of coffee consumption with femoral neck and total hip BMD.

Outcome	Visit	Model 1				Model 2			
		No	Yes	Difference (95% CI)	*p* Value	No	Yes	Difference (95% CI)	*p* Value
LS means of femoral neck BMD (g/cm^2^)	2	0.654	0.654	0.000 (−0.002, 0.001)	0.6069	0.650	0.649	−0.001 (−0.003, 0.001)	0.5924
	4	0.633	0.633	0.000 (−0.002, 0.002)	0.9708	0.630	0.629	−0.001 (−0.003, 0.002)	0.6394
	5	0.624	0.625	0.001 (−0.001, 0.003)	0.4816	0.622	0.623	0.001 (−0.002, 0.003)	0.5098
	6	0.617	0.618	0.001 (−0.002, 0.003)	0.8268	0.616	0.617	0.001 (−0.002, 0.003)	0.6465
LS means of total hip BMD (g/cm^2^)	2	0.764	0.764	0.000 (−0.001, 0.002)	0.7576	0.757	0.757	0.000 (−0.001, 0.002)	0.6981
	4	0.736	0.737	0.001 (−0.001, 0.003)	0.3411	0.733	0.734	0.001 (−0.001, 0.003)	0.5053
	5	0.725	0.726	0.001 (−0.001, 0.003)	0.3841	0.724	0.725	0.001 (−0.001, 0.003)	0.4745
	6	0.716	0.715	−0.002 (−0.004, 0.001)	0.1548	0.716	0.716	0.000 (−0.003, 0.002)	0.8666

**Model 1**: Adjusted for coffee consumption, visit, visit × coffee interaction, and baseline BMD (measured at visit 2 for each outcome site). **Model 2**: Additionally adjusted for time-dependent covariates, including age, BMI, current smoking status, Charlson Comorbidity Index, and the other beverage exposure (e.g., tea consumption adjusted for coffee consumption), as well as time-independent covariates, including age at menopause, lifetime alcohol use, average weekly calcium intake, average weekly protein intake, weighted lifetime physical activity, current oral estrogen use, and current steroid pill use. **LS mean**: least squares mean.

**Table 3:** Associations of tea consumption with femoral neck and total hip BMD.

Outcome	Visit	Model 1 for Tea Consumption				Model 2 for Tea Consumption			
		No	Yes	Difference (95% CI)	*p* Value	No	Yes	Difference (95% CI)	*p* Value
LS means of femoral neck BMD (g/cm^2^)	2	0.654	0.654	0.000 (−0.002, 0.002)	0.8217	0.649	0.650	0.001 (−0.002, 0.003)	0.5590
	4	0.632	0.633	0.002 (−0.001, 0.004)	0.1385	0.629	0.631	0.001 (−0.001, 0.004)	0.2169
	5	0.624	0.625	0.000 (−0.002, 0.003)	0.7296	0.622	0.623	0.000 (−0.003, 0.002)	0.9381
	6	0.618	0.617	−0.001 (−0.004, 0.001)	0.2928	0.618	0.615	−0.002 (−0.005, 0.001)	0.1341
LS means of total hip BMD (g/cm^2^)	2	0.764	0.764	0.000 (−0.002, 0.002)	0.8951	0.756	0.757	0.001 (−0.001, 0.003)	0.5640
	4	0.736	0.738	0.001 (−0.001, 0.003)	0.1975	0.733	0.734	0.002 (−0.001, 0.004)	0.1407
	5	0.726	0.726	0.000 (−0.002, 0.002)	0.6571	0.724	0.725	0.000 (−0.002, 0.002)	0.8541
	6	0.715	0.717	0.002 (0.000, 0.004)	0.0354	0.715	0.718	0.003 (0.000, 0.005)	0.0260

**Model 1**: Adjusted for coffee consumption, visit, visit × coffee interaction, and baseline BMD (measured at visit 2 for each outcome site). **Model 2**: Additionally adjusted for time-dependent covariates, including age, BMI, current smoking status, Charlson Comorbidity Index, and the other beverage exposure (e.g., tea consumption adjusted for coffee consumption), as well as time-independent covariates, including age at menopause, lifetime alcohol use, average weekly calcium intake, average weekly protein intake, weighted lifetime physical activity, current oral estrogen use, and current steroid pill use. **LS mean**: least squares mean.

## Data Availability

The original data presented in the study are openly available in SOF online, at https://sofonline.ucsf.edu (accessed on 9 October 2025). To access data, you must create an account and electronically sign a Data Use Agreement.

## Abbreviations

The following abbreviations are used in this manuscript:

BMD	Bone mineral density
CCI	Charlson comorbidity index
DALY	Disability-adjusted life years
MAR	Missing at random
LS	Least squares
SOF	Study of osteoporotic fractures
YLD	Years lived with disability

## Appendix A

### Appendix A.1

Sensitivity analyses extending follow up visits to visit 8 and visit 9. Associations of coffee consumption with femoral and total hip BMD.

**Table A1:** Associations of coffee consumption with femoral neck and total hip BMD.

Outcome	Visit	Model 1				Model 2			
		No	Yes	Difference (95% CI)	*p* Value	No	Yes	Difference (95% CI)	*p* Value
LS means of femoral neck BMD (g/cm^2^)	2	0.656	0.655	0.000 (−0.002, 0.002)	0.6859	0.651	0.650	0.000 (−0.003, 0.002)	0.7187
	4	0.634	0.634	0.000 (−0.002, 0.002)	0.9345	0.631	0.631	−0.001 (−0.003, 0.002)	0.6725
	5	0.626	0.627	0.001 (−0.001, 0.003)	0.4246	0.623	0.624	0.001 (−0.002, 0.004)	0.4480
	6	0.619	0.619	0.000 (−0.002, 0.003)	0.7388	0.617	0.618	0.001 (−0.002, 0.004)	0.5827
	8	0.611	0.615	0.003 (0.000, 0.006)	0.0565	0.613	0.616	0.003 (−0.001, 0.007)	0.0921
	9	0.612	0.600	−0.012 (−0.018, −0.005)	0.0010	0.620	0.609	−0.011 (−0.019, −0.003)	0.0071
LS means of total hip BMD (g/cm^2^)	2	0.766	0.766	0.000 (−0.001, 0.002)	0.6758	0.756	0.757	0.001 (−0.001, 0.003)	0.5623
	4	0.738	0.740	0.001 (−0.001, 0.003)	0.2275	0.733	0.734	0.001 (−0.001, 0.003)	0.3717
	5	0.727	0.728	0.001 (−0.001, 0.003)	0.2608	0.725	0.726	0.001 (−0.001, 0.004)	0.2916
	6	0.718	0.717	−0.001 (−0.003, 0.001)	0.3545	0.717	0.717	0.000 (−0.002, 0.003)	0.8971
	8	0.703	0.706	0.003 (0.000, 0.006)	0.0283	0.709	0.712	0.003 (0.000, 0.006)	0.0697
	9	0.706	0.704	−0.002 (−0.008, 0.005)	0.6005	0.723	0.721	−0.002 (−0.009, 0.005)	0.5435

Model 1: Adjusted for coffee consumption, visit, visit × coffee interaction, and baseline BMD (measured at visit 2 for each outcome site). Model 2: additionally adjusted for time dependent covariates, age, BMI, current smoking status, Charlson comorbidity index, as well as the other beverage exposure (e.g., tea consumption adjusted for coffee consumption) and time independent covariates age at menopause, lifetime alcohol use, average calcium consumed per week, average protein consumed per week, weighted lifetime physical activity, lifetime oral estrogen used was also included.

### Appendix A.2

Sensitivity analyses extending follow up visits to visit 8 and visit 9. Associations of tea consumption with femoral and total hip BMD.

**Table A2:** Associations of tea consumption with femoral neck and total hip BMD.

Outcome	Visit	Model 1				Model 2			
		No	Yes	Difference (95% CI)	*p* Value	No	Yes	Difference (95% CI)	*p* Value
LS means of femoral neck BMD (g/cm^2^)	2	0.655	0.656	0.000 (−0.002, 0.003)	0.7318	0.650	0.651	0.001 (−0.002, 0.003)	0.4955
	4	0.634	0.635	0.002 (0.000, 0.004)	0.1240	0.630	0.632	0.002 (−0.001, 0.004)	0.1855
	5	0.626	0.626	0.000 (−0.002, 0.003)	0.8256	0.624	0.624	0.000 (−0.003, 0.003)	0.9554
	6	0.620	0.619	−0.001 (−0.004, 0.002)	0.4261	0.619	0.617	−0.002 (−0.005, 0.001)	0.2360
	8	0.612	0.615	0.004 (0.000, 0.007)	0.0286	0.613	0.616	0.003 (−0.001, 0.007)	0.1139
	9	0.604	0.605	0.001 (−0.006, 0.008)	0.7669	0.613	0.614	0.001 (−0.007, 0.009)	0.8361
LS means of total hip BMD (g/cm^2^)	2	0.766	0.767	0.000 (−0.002, 0.002)	0.7088	0.756	0.757	0.001 (−0.001, 0.003)	0.3118
	4	0.738	0.740	0.002 (0.000, 0.004)	0.1383	0.733	0.735	0.002 ( 0.000, 0.004)	0.0683
	5	0.728	0.728	0.001 (−0.002, 0.003)	0.6265	0.725	0.726	0.001 (−0.002, 0.003)	0.6044
	6	0.717	0.719	0.003 (0.000, 0.005)	0.0329	0.716	0.719	0.003 (0.001, 0.006)	0.0159
	8	0.703	0.707	0.005 (0.001, 0.008)	0.0040	0.709	0.714	0.004 (0.001, 0.007)	0.0187
	9	0.704	0.706	0.002 (−0.005, 0.008)	0.5952	0.722	0.720	−0.001 (−0.009, 0.006)	0.7022

Model 1: Adjusted for tea consumption, visit, visit × tea interaction, and baseline BMD (measured at visit 2 for each outcome site). Model 2: additionally adjusted for time dependent covariates, age, BMI, current smoking status, Charlson comorbidity index, as well as the other beverage exposure (e.g., tea consumption adjusted for coffee consumption) and time independent covariates age at menopause, lifetime alcohol use, average calcium consumed per week, average protein consumed per week, weighted lifetime physical activity, lifetime oral estrogen used was also included.
